# The effect of Curcumin on metabolic parameters and androgen level in women with polycystic ovary syndrome: a randomized controlled trial

**DOI:** 10.1186/s12902-023-01295-5

**Published:** 2023-02-15

**Authors:** Niloofar Ghanbarzadeh-Ghashti, Solmaz Ghanbari-Homaie, Elnaz Shaseb, Shamsi Abbasalizadeh, Mojgan Mirghafourvand

**Affiliations:** 1 Students’ research committee, Nursing and Midwifery Faculty, Tabriz University of Medical Sciences, Tabriz, Iran; 2 Department of Midwifery, Faculty of Nursing and Midwifery, Tabriz University of Medical Sciences, Tabriz, Iran; 3 Department of Clinical Pharmacy, Faculty of Pharmacy, Tabriz University of Medical Sciences, Tabriz, Iran; 4 Women’s Reproductive Health Research Center, Tabriz University of Medical Sciences, Tabriz, Iran; 5 Social Determinants of Health Research Center, Faculty of Nursing and Midwifery, Tabriz University of Medical Sciences, Tabriz, Iran

**Keywords:** Curcumin, Hirsutism, Polycystic ovary syndrome, Metabolic, Hormone

## Abstract

**Background:**

Considering the high prevalence of polycystic ovary syndrome (PCOS) in women of reproductive age and the metabolic disorders associated with it, this study was conducted to determine the effects of curcumin on metabolic indices and androgen level (primary outcomes), and menstruation characteristics, and hirsutism (secondary outcomes) in women with PCOS.

**Methods:**

This triple-blind randomized controlled trial was conducted on women with PCOS who visited the health centers at Eslamshahr County (Tehran Province-Iran) from 2020 to 2022. The participants were allocated into two groups (curcumin and placebo) using block randomization method. The treatment group received two 500 mg edible curcumin tablets together at the same time per day for twelve weeks while the control group received placebo tablets similar to curcumin. Biochemical parameters such as Fasting Blood Insulin (FBI), Fasting Blood Sugar (FBS), triglyceride, total cholesterol, Low Density Lipoprotein- cholesterol (LDL-C), High Density Lipoprotein- cholesterol (HDL-C) were measured before intervention and then 3 months after the intervention. Sex Hormone Binding Globulin (SHBG) and testosterone serum levels were measured 3 months after the intervention. Questionnaires regarding the menstrual cycle characteristics and the Ferriman–Gallwey score were also filled for evaluating hirsutism before the intervention as well as 3 months after the intervention. The independent t-test, Mann-Whitney U test, and ANCOVA were used to analyze the data.

**Results:**

There was no statistically significant difference between the two groups in terms of socio-demographic and the baseline levels of measured outcomes. After 12 weeks of intervention, the mean serum FBS levels in the curcumin group were significantly lower than in the placebo group (mean difference: 6.24; 95%confidence interval: -11.73 to -0.76; P = 0.027) but there was no significant difference between the two groups in terms of triglyceride (P = 0.351), cholesterol (P = 0.528), LDL (P = 0.064), HDL (P = 0.306), FBI (p = 0.929), SHBG (p = 0.682), and testosterone (p = 0.133) serum levels. After the intervention, amenorrhea and oligomenorrhea frequency in the curcumin group was significantly lower than in the placebo group (13% vs. 22%, P = 0.038). There was no significant difference in terms of duration of menstruation (P = 0.286) and hirsutism (P = 0.630) between the two groups.

**Conclusion:**

Curcumin decreased FBS levels and improved menstruation characteristics (amenorrhea, oligomenorrhea, and menstrual irregularities) in women with PCOS but did not affect other metabolic, hormonal, and hirsutism indices. More studies using a larger sample size are required for a definitive conclusion.

**Trial registration:**

Iranian Registry of Clinical Trials (IRCT): IRCT20120718010324N51 Date of registration: 30/11/2019. URL: https://en.irct.ir/user/trial/40597/view; Date of first registration: 30/11/2020.

## Background

Polycystic ovary syndrome (PCOS), also known as metabolic syndrome or insulin resistance syndrome [1, 2], is a common endocrine and metabolic disorder among women [3] and is a series of symptoms related to hormonal imbalance [4]. This hormonal imbalance causes irregularities in ovulation that could lead to the formation of small cysts in the ovary [5]. This syndrome is the most common hormonal disorder in women [6] and is seen in 4–6% of women of reproductive age. Studies suggest an increase in this syndrome [6]. The prevalence of PCOS among Iranian women is reported to be between 7.1 and 14.6% [7].

Many of the systems in the body are affected by PCOS which leads to several complications such as menstrual disorders, infertility, hirsutism, acne, obesity and metabolic syndrome [8], increase in LH levels, slight decrease or lack of change in FSH levels, a slight increase in prolactin, increase in estradiol and estrogen levels, possible increase in dehydroepiandrosterone sulfate (DHEAS), androstenedione, and testosterone, and decrease in sex hormone-binding globulin (SHBG) levels [9]. Metabolic disorders are seen in women with PCOS include resistance to insulin, hyperinsulinemia, and dyslipidemia (decrease in High Density Lipoprotein- cholesterol (HDL-C), increase in total cholesterol, Low Density Lipoprotein- cholesterol (LDL-C), and triglycerides), so we can say that women with PCOS are prone to type 2 diabetes and cardiovascular diseases [10]. Insulin’s role in PCOS is to regulate the activities of ovary and liver enzymes that have a role in the production of androgen and low-grade inflammation, respectively. Reportedly, insulin resistance usually leads to problems such as dyslipidemia as well as cardiovascular and metabolic diseases [11].

PCOS pathogenesis are still unknown but studies show that PCOS has a complicated and multi-factor cause that stems from the interaction of genetic, environmental, and intrauterine factors [12]. Currently, there is no cure for PCOS and as treatment, women with PCOS are only recommended to change their lifestyle [13]. Most of these interventions focus on changing eating habits, physical activity, and losing weight [14, 15]. However, new studies in the field of medicinal herbs and complementary therapy have shown promising results in curing PCOS.

Curcumin is a polyphenol derived from the curcuma longa species and traditionally can be found in Asian food [16]. Recently curcumin was studied as supportive therapy for a wide range of diseases such as type 2 diabetes which is one of the complications of PCOS [17]. Based on a study on animals, curcumin can be effective in improving insulin sensitivity and decreasing CRP and IL-6 levels [18]. Moreover, it was reported in a study on humans that the edible intake of curcumin led to a significant decrease in FBS, HOMA-IR, HbA1c, triglycerides, and total cholesterol levels in patients with metabolic diseases.

As seen in animal experiments, the use of curcumin can lead to a decrease in insulin resistance (one of the key features of PCOS phenotype), a decrease in blood’s inflammatory factors such as CRP and IL-6 [18] (which are high in people with PCOS and have a direct relationship with insulin sensitivity [19], a decrease in FBS and cholesterol levels in people with metabolic disorders (complications of PCOS) [20], but the administration of curcumin can lead to improvement in insulin sensitivity, cholesterol levels and metabolic factors in people with PCOS as well. So, this study was conducted to determine the effects of curcumin on metabolic indices and androgen level (primary outcomes), menstruation characteristics and hirsutism (secondary outcomes) in women with PCOS.

## Methods

### Study design and setting

This study was a superiority randomized controlled trial with two-parallel arms. The participants, researcher, and analyst in this study were blinded to the intervention received by the participants. This study was conducted in the health centers at Eslamshahr County (Tehran Province-Iran) from 2020 to 2022.

### Eligibility criteria

The study was conducted on women aged 18 to 45 with PCOS and a BMI of 18.5 to 40. The exclusion criteria were as follows: taking vitamins, contraceptives, hormonal drugs, minerals, and omega-3 during the study and 3 months before intervention; having other metabolic diseases such as diabetes and androgenic disorders such as congenital adrenal hyperplasia (CAH) or androgen-producing glands; thyroid gland diseases; Cushing syndrome; pregnancy or breastfeeding; prior surgery on one or both ovaries; smoking and alcohol consumption.

### Sampling

Sampling started after the approval of the ethics committee of Tabriz University of Medical Sciences (Ethics code: IR.TBZMED.REC.1398.017) and registering the study in Iranian Registry of Clinical Trials (IRCT) (Code: IRCT20120718010324N51). The researcher attended at the health centers in the Eslamshahr city and searched for women with eligible criteria and willingness to participate in the study. They were surveyed based on Rotterdam criteria (the presence of two of the three criteria is enough to diagnose PCOS) which include oligomenorrhea (i.e., a menstrual cycle that lasts more than 35 days or having four to nine cycles each year) [21] or lack of ovulation, clinical and laboratory signs and symptoms of hyperandrogenism, and polycystic ovary.

After PCOS was confirmed, written consent is obtained in case of the patient’s willingness to participate in the study and questionnaires regarding socio-demographic and menstrual cycle characteristics were completed. A food consumption frequency questionnaire was also provided to the participants to record their average annual food consumption. The hirsutism score of the participants was determined by the researcher using Ferriman-Gallwey criteria as well as their weight and height to determine their BMI before intervention were measured. A checklist was given to the participants to remind them of their daily medication use, and they were contacted monthly to ensure that they were taking the medication.

The participants’ biochemical parameters including Fasting Blood Insulin (FBI), Fasting Blood Sugar (FBS), triglyceride, total cholesterol, LDL-C, HDL-C were measured before intervention and then 3 months after. The participants were directed to Ava laboratory in Eslamshahr for those measurements. Venous sampling was used with a 5 ml syringe by the laboratory personnel to extract 5 ml of blood from the brachial region. The test results were provided to the researcher. Three months after the intervention, hormonal tests (testosterone and SHBG) were measured as well. All blood tests were conducted at a certain stage of the menstrual cycle (follicular phase). Insulin resistance index (HOMA-IR) was calculated based on FBS and FBI. The participants were to refrain from using hormonal drugs, and supplements, having specific diets or doing heavy sports during the study.

### Random allocation & intervention

The participants were allocated into two groups (curcumin and placebo) using block randomization with a ratio of 1:1. Identical black opaque bottles numbered consecutively (in order of allocation) were used to conceal what was allocated to each participant. The bottles were prepared by a third party that had no role in the sampling or gathering and analysis of data. The curcumin and placebo were produced by Dineh Iran pharmaceutical company (Qazvin, Iran). The intervention group received two 500 mg tablets of edible curcumin together at the same time per day for twelve weeks while the control group received placebo tablets similar to curcumin. Placebo and curcumin tablets were identical in terms of weight, shape, color and smell.

### Data collection tools

Questionnaires for socio-demographic information, a checklist for recording tests, a menstrual cycle characteristics questionnaire, and the Ferriman–Gallwey scale were used to collect the data. The food frequency questionnaire (FFQ) was used to measure the nutritional status of the participants.

The menstrual cycle characteristics questionnaire includes questions about the average interval of menstrual cycles and the average duration of bleeding in the last three months which are asked twice, once at the beginning of the study and then three months after the intervention.

The Ferriman–Gallwey scale is also used in the same two time periods to evaluate hirsutism. This scale evaluates the state of hair growth in 9 areas of the body and assigns a score between zero and four according to the Likert scale. Zero means no hair and four means excessive hair growth. The highest possible score for any one participant in this questionnaire would be 36 and the lowest score would be zero [22].

The FFQ used in this study included 67 types of food which were used to gather information about the intake of each food. The participants were asked about the frequency and amount of intake of each food during a day, week, and month. The data was eventually summarized into “how many times in the week the participant ate each type of food”, “how many grams of it was eaten each time”, and “how many grams of it was eaten each week”.

To assess adherence to treatment, the participants were requested to record the drug use in a drug use checklist.

### Sample size

The sample size was determined based on the testosterone levels using G*Power software. Considering the data from the Gholizadeh Shamasbi study [23]; a 25% decrease in mean FBS levels; and the following values, M1 = 80.68; Sd1 = Sd2 = 12.3, M2 = 67.6; power = 95% and the two-sided α = 0.05, the sample size was calculated equal with 25 people for each group. With this calculated sample size, the trial has > 95% power for other primary outcomes except of testosterone variable.

### Statistical analysis

After gathering the data, SPSS software version 24 was used for data analysis. Descriptive statistics such as mean (standard deviation) were used for quantitative data and number (percentage) was used for qualitative data. Median (percentile 25 to percentile 75) was used for quantitative data that had an abnormal distribution. Kolmogorov-Smirnov test was used to evaluate the normality of the data. Variables such as fasting insulin and HOMA-IR before and after intervention, testosterone, SHBG, hirsutism before and after intervention, and the duration of menstruation had an abnormal distribution while other variables had a normal distribution. An independent t-test was used for an intergroup comparison of variables with a normal distribution before intervention and after the intervention, ANCOVA test was used with a control of baseline values. Mann-Whitney U test was used for an intergroup comparison of variables with an abnormal distribution before and after the intervention. The paired samples t-test and Wilcoxon signed ranks test were used for intra-group comparisons of outcomes with normal and abnormal distribution, respectively.

To analyze the FFQ, the nutrient units were converted to grams per day using the book “The Manual for Household Measures”. Also, any food or drink was coded and recorded in Nutritionist IV software which was adjusted for Iranian food to evaluate the amount of energy and nutrients received by the participants. The intake of energy, macronutrients, and micronutrients of each participant was also determined. All of the analysis was done base on an intention to treat. P < 0.05 was considered as significant.

## Results

**Fig. 1:**
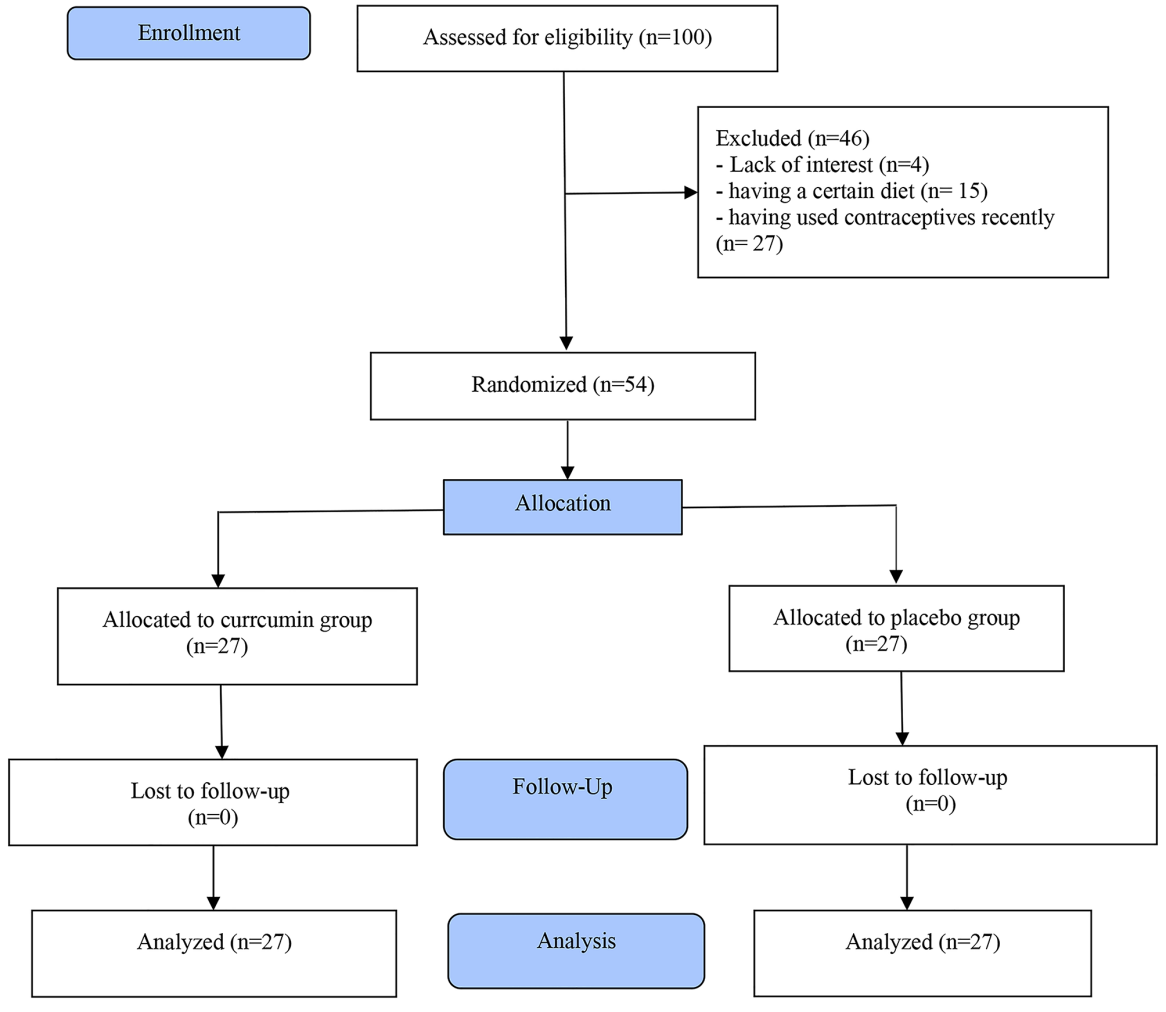
Study flow chart

Between 2019 and 2022 about 100 people were evaluated in terms of having the necessary criteria. Four did not enter the study for lack of interest, 15 for having a certain diet, and 27 for having used contraceptives recently. In the end, the data from 27 people in the intervention group and 27 people in the control group were analyzed (Fig. 1). The percentage of adherence to therapy in the both groups was 100%.

The average age of women in the curcumin group was 28.44 (SD: 7.61) years while the average age of women in the placebo group was 30.85 (SD: 8.63) years. The BMI score of the curcumin group was 27.34 (SD: 5.42) kg/m^2^ and the placebo group was 26.41 (SD: 4.21) kg/m^2^. Most women in both groups were nulliparous (74% in the curcumin group and 63% in the placebo group). There was no significant statistical difference between the curcumin and placebo groups in terms of socio-demographic and obstetrics characteristics. There was no statistically significant difference between the two groups in terms of intake of nutrients such as energy, cholesterol, protein, saturated fat, carbohydrate, total fat, and fiber as well (Table 1).

**Table 1:** Socio-demographic characteristics and nutritional values by study groups

Characteristic	Curcumin n = 27	Placebo n = 27	p-value
	mean (SD*)	**mean (SD** ^*^ **)**	
**Age** (Year)	28.44 (7.617)	30.85 (8.632)	0.282^†^
**BMI** (Kg/m2 )	27.34 (5.42)	26.41 (4.21)	0.482^†^
**Total fat** (Gram/day)	74.41 (37.8)	77.96 (27.54)	0.695^†^
**Fiber** (Gram/day)	25.37 (12.44)	22.25 (9.12)	0.299^†^
**Cholesterol** (Gram/day)	341.79 (408.94)	251.54 (148.27)	0.869^€^
**Calories** (Kcal/day)	1897.40 (1054.13)	1896.74 (623.09)	0.392^€^
**Protein** (Gram/day)	71.05 (56.53)	63.45 (25.31)	0.672^€^
**Saturated fatty acids** (Gram/day)	23.57 (12.31)	25.31 (9.48)	0.303^€^
**Carbohydrates** (Gram/day)	239.82 (133.19)	244.72 (87.78)	0.355^€^
	**Number (Percent)**	**Number (Percent)**	
**Marital status**			0.083^§^
Single	12 (44.4)	6 (22.2)	
Married	15 (55.6)	21 (77.8)	
**Education**			0.355^‡^
Under Diploma	0 (0)	1 (3.7)	
Diploma	12 (44.4)	8 (29.6)	
University	15 (55.6)	18 (66.7)	
**Job**			0.535^§^
Housewife	21 (77.8)	19 (70.4)	
Employed	6 (22.2)	8 (29.6)	
**Spouse’s job**			0.202^¥^
Self employed	7 (25.9)	12 (46.2)	
Employed	8 (29.6)	7 (26.9)	
Worker	0 (0)	1 (3.8)	
**Gravid**			0.773^¥^
0	20 (74.1)	16 (59.3)	
1–2	5 (16.5)	11 (29.6)	
3–4	2 (7.4)	3 (11.1)	
**Parity**			0.057^¥^
0	20 (74.1)	17 (63)	
1–2	5 (18.5)	9 (33.3)	
3	2 (7.4)	1 (3.7)	
**Abortion**			0.704^¥^
0	24 (88.9)	22 (81.5)	
1	3 (11.1)	5 (18.5)	

* Standard Deviation; ^†^ Independent t-test; ^‡^ Chi-square for trend; ^§^ Chi-square; ^¥^ Fisher’s Exact Test; ^€^ Mann-Whitney U

## Primary outcomes

The mean (SD) serum FBS level before intervention was 93.92 (9.12) in the curcumin group and 91.33 (9.40) in the placebo group so there was no statistically significant difference between the two groups (P = 0.314). After 12 weeks of intervention, the mean (SD) serum FBS level in the curcumin group was 90.38 (10.69) and in the placebo group it was 95.22 (11.22) which is significantly higher than the curcumin group (Mean Difference (MD): -6.24; 95% Confidence Interval (95% CI): -11.73 to -0.76; P = 0.027).

The mean (SD) serum triglyceride level before intervention was 110.81 (47.11) in the curcumin group and 137.19 (43.20) in the placebo group which means a statistically significant difference between the two groups (P = 0.038). After 12 weeks of intervention, the mean (SD) serum triglyceride level in the curcumin group was 128.02 (6.13) and in the placebo group it was 136.27 (6.00) which means no statistically significant difference between the two groups (MD: -8.25; 95% CI: -25.85 to 9.35; P = 0.351).

The mean (SD) serum cholesterol level before intervention was 158.46 (32.9) in the curcumin group and 162.59 (34.97) in the placebo group so there was no statistically significant difference between the two groups (P = 0.660). After 12 weeks of intervention, the mean (SD) serum cholesterol level in the curcumin group was 165.85 (37.69) and in the placebo group it was 174.19 (40.93) which means no statistically significant difference between the two groups (MD: -6.25; 95% CI: -26.13 to 13.57; P = 0.528).

The mean (SD) serum LDL level before intervention was 88.77 (25.33) in the curcumin group and 91.11 (27.99) in the placebo group so there was no statistically significant difference between the two groups (P = 0.751). After 12 weeks of intervention, the mean (SD) serum LDL level in the curcumin group was 89.96 (30.92) and in the placebo group it was 105.70 (29.33) which means no statistically significant difference between the two groups (MD: -14.81; 95% CI: -30.52 to 0.91; P = 0.064).

The mean (SD) serum HDL level before intervention was 50.12 (10.89) in the curcumin group and 105.70 (29.33) in the placebo group so there was no statistically significant difference between the two groups (P = 0.614). After 12 weeks of intervention, the mean (SD) serum HDL level in the curcumin group was 53.77 (13.15) and in the placebo group it was 51.56 (9.74) which means no statistically significant difference between the two groups (MD: 3.22; 95% CI: -3.04 to 9.48; P = 0.306).

The mean (SD) serum fasting insulin level before intervention was 12.54 (7.56) in the curcumin group and 9.76 (7.25) in the placebo group so there was no statistically significant difference between the two groups (P = 0.135). After 12 weeks of intervention, the mean (SD) serum fasting insulin level in the curcumin group was 9.50 (7.02) and in the placebo group it was 10.11 (9.30) which means no statistically significant difference between the two groups (P = 0.929).

The mean (SD) HOMA-IR before intervention was 2.88 (1.99) in the curcumin group and 2.16 (1.43) in the placebo group so there was no statistically significant difference between the two groups (P = 0.229). After 12 weeks of intervention, the mean (SD) serum HOMA-IR in the curcumin group was 2.11 (1.79) and in the placebo group it was 2.40 (2.11) which means no statistically significant difference between the two groups (P = 0.736).

After the intervention, the mean (SD) serum testosterone level in the curcumin group was 0.64 (0.39) and in the placebo group it was 0.77 (0.36) which means no statistically significant difference between the two groups (0.133). After the intervention, the mean (SD) serum SHBG level in the curcumin group was 57.11 (38.57) and in the placebo group it was 60.67 (40.87) which means no statistically significant difference between the two groups (P = 0.682).

The intra-group comparison results showed that there was statistical significant difference between before and after intervention in terms of fasting insulin level (P = 0.005) and HOMA-IR (P = 0.005) in the curcumin group. Also, the post-intervention FBS level increased significantly compared to pre-intervention level in the placebo group (P = 0.037) (Table 2).

**Table 2:** Comparison of the metabolic indices levels by study groups

Variable	Curcumin (n = 27) Mean (SD^*^)	Placebo (n = 27) Mean (SD^*^)	Mean Difference (95% Confidence Interval)	P-value
**Fasting blood sugar** (mg/dl)				
Before intervention	93.92 (9.12)	91.33 (9.40)	2.59 (-2.52 to 7.70)	0.314^†^
After intervention	90.38 (10.69)	95.22 (11.226)	-6.24 (-11.73 to -0.76)	0.027^‡^
Intra-group comparison (P-value^* *^)	0.144	0.037		
**Triglyceride** (mg/dl)				
Before intervention	110.81 (47.11)	137.19 (43.20)	-26.38 (-51.29 to -1.47)	0.038^†^
After intervention	128.02 (6.13)	136.27 (6.00)	-8.25 (-25.85 to 9.35)	0.351^‡^
Intra-group comparison (P-value^* *^)	0.214	0.202		
**Cholesterol** (mg/dl)				
Before intervention	158.46 (32.94)	162.59 (34.97)	-4.13 (-22.88 to 14.62)	0.660^†^
After intervention	165.85 (37.69)	174.19 (40.93)	-6.28 (-26.13 to 13.57)	0.528
Intra-group comparison (P-value^* *^)	0.167	0.228		
**LDL-C** (mg/dl)				
Before intervention	88.77 (25.33)	91.11 (27.99)	-2.342 (-17.08 to 12.40)	0.751^†^
After intervention	89.96 (30.92)	105.70 (29.33)	-14.81 (-30.52 to 0.91)	0.064^‡^
Intra-group comparison (P-value^* *^)	0.783	0.070		
**HDL-C** (mg/dl)				
Before intervention	50.12 (10.89)	51.56 (9.74)	-1.44 (-7.13 to 4.25)	0.614^†^
After intervention	53.77 (13.15)	51.56 (9.74)	3.22 (-3.04 to 9.48)	0.306^‡^
Intra-group comparison (P-value^* *^)	0.198	0.873		
**Fasting Insulin** (mg/dl)				
Before intervention	12.54 (7.56)	9.76 (7.25)	-	0.135^§^
After intervention	9.50 (7.02)	10.11 (9.30)	-	0.929^§^
Intra-group comparison (P-value^††^)	0.005	0.760		
**HOMA-IR**				
Before intervention	2.88 (1.99)	2.16 (1.43)		0.229^§^
After intervention	2.11 (1.79 )	2.40 (2.11)		0.736^§^
Intra-group comparison (P-value^††^)	0.005	0.792		
**Testosterone** (After intervention) (ng/dl)	0.64 (0.39)	0.77 (0.36)	-	0.133^§^
**SHBG**^¥^ (After intervention) (µg/dl)	57.11 (38.57)	60.67 (40.87)	-	0.682^§^

* Standard Deviation; ^†^ Independent t-test; ^‡^ ANCOVA; ^§^ Mann-Whitney U; ^¥^ Sex Hormone Binding Globulin; ^* *^ Paired samples t-test; ^††^ Wilcoxon signed ranks test

## Secondary outcomes

Amenorrhea (i.e., cessation of previously regular menses for three months or previously irregular period for six months) [24] frequency before intervention was %26.98 in the curcumin group and %37.0 in the placebo group while oligomenorrhea frequency was %63.0 in the curcumin group and % 59.3in the placebo group, so there was no statistically significant difference between the two groups. After the intervention, the amenorrhea frequency was %11.1 in the curcumin group and %29.68 in the placebo group while the oligomenorrhea frequency was %37.0 in the curcumin group and %51.9 in the placebo group, so there was a statistically significant difference between the two groups in terms of menstruation characteristics (P = 0.038). The median (Percentile 25 to 75) length of menstruation before intervention was 4.0 (2.0 to 5.0) in the curcumin group and 5.0 (3.0 to 5.0) in the placebo group, so there was no significant difference between the two. The median (Percentile 25 to 75) length of menstruation after the intervention was 4.0 (0.0 to 5.0) in the curcumin group and 4.0 (2.0 to 5.0) in the placebo group, so there was no statistically significant difference between the two groups (P = 0.286) (Table 3).

**Table 3:** Comparison of the menstrual characteristics among study groups

Characteristics	Curcumin (n = 27)	Placebo (n = 27)	P-value
**Interval between menstrual cycles**	**Number (Percent)**	**Number (Percent)**	
**Before intervention**			0.825^*^
Amenorrhea	8 (26.9)	10 (37.0)	
Regular	2 (7.4)	1 (3.7)	
Oligomenorrhea	17 (63.0)	16 (59.3)	
**After intervention**			0.038^*^
Amenorrhea	3 (11.1)	8 (29.6)	
Regular	13 (48.1)	5 (18.5)	
Oligomenorrhea	10 (37.0)	14 (51.9)	
**Duration of menstrual cycles**	**Median (Per 25 to Per 75** ^**‡**^ **)**	**Median (Per 25 to Per 75** ^**‡**^ **)**	
Before intervention	4.0 (2.0 to 5.0)	5.0 (3.0 to 5.0)	0.715^†^
After intervention	4.0 (0.0 to 5.0)	4.0 (2.0 to 5.0)	0.286^†^
Intra-group comparison (P-value^§^)	0.076	0.461	

* Chi-square; ^†^ Mann-Whitney U; ^‡^ Percentile 25 to Percentile 75; ^§^ Wilcoxon signed ranks test

The mean hirsutism score (SD) before intervention was 11.85 in the curcumin group (4.63) and 12.19 (3.65) in the placebo group so there was no statistically significant difference between the two groups (P = 0.602). The mean (SD) hirsutism score after intervention in the curcumin group was 11.92 (4.71) and 12.19 (3.65) in the placebo group, which means no statistically significant difference between the two groups (P = 0.630) (Table 4).

**Table 4:** Comparison of hirsutism score between study groups

Hirsutism	Curcumin (n = 27)	Placebo (n = 27)	P-value^†^
	Median (Per 25 to Per 75^‡^)	Median (Per 25 to Per 75^‡^)	
Before intervention	11.0 (9.0 to 13.0)	11.0 (9.0 to 15.0)	0.602
After intervention	11.0 (9.0 to 13.2)	11.0 (9.0 to 15.0)	0.630
Intra-group comparison (P-value^§^)	1.000	1.000	

^‡^ Percentile 25 to Percentile 75; ^†^ Mann-Whitney U; ^§^ Wilcoxon signed ranks test

## Discussion

According to the study results, administrating curcumin only led to a decrease in FBS levels and did not significantly affect other metabolic indices (triglyceride, cholesterol, LDL-C, HDL-C, insulin, testosterone and SHBG). Regarding the amenorrhea and oligomenorrhea frequency, the numbers in the curcumin group were significantly lower than in the placebo group but there was no statistically significant difference between the two groups in terms of length of menstruation. Also, curcumin had no significant positive effect on hirsutism in women with PCOS.

In the present study, administrating curcumin led to a decrease in FBS, FBI and HOMA-IR in the curcumin group. Our results were partly consistent with those of previous studies; in a randomized clinical trial, 60 individuals with PCOS were randomly divided into a curcumin and placebo groups and received 500 mg of curcumin or placebo tablets for 12 weeks. Generally administrating curcumin for 12 weeks to women with PCOS had positive effects on body weight, controlling the blood sugar, serum lipids (except triglycerides), very low-density lipoprotein (VLDL-cholesterol) levels, and peroxisome proliferator-activated receptor gamma (PAR-g) and low-density lipoprotein receptor (LDLR) gene expression [25]. In another randomized trial, 67 individuals with PCOS received 500 mg curcumin or placebo tablets three times a day, for 12 weeks. According to the study results, plasma fasting glucose levels, as well as dehydroepiandrosterone, were decreased significantly in the curcumin group in comparison to the placebo group [26].

In a systematic review, the effect of curcumin on glycemic control and lipid profile was examined. Based on the result of the reviewed studies, curcumin improved fasting glucose levels, insulin, and homeostasis model assessment of insulin resistance (HOMA-IR) in a significant way. Moreover, high-density lipoprotein and cholesterol levels were significantly improved. But it did not have any significant effect on improving low-density lipoprotein and triglyceride levels [27]. This is following the results of our study which showed that curcumin improved fasting glucose levels but had no significant effect on triglyceride and LDL.

Curcumin decreases blood sugar through different mechanisms such as increasing glucose and glycolysis uptake and glycogen synthesis in skeletal muscles or decreasing gluconeogenesis in the liver [28]. Furthermore, curcumin was shown to be able to increase glucose uptake by increasing phosphorylation in AMP-activated protein kinase (AMPK) [29]. Curcumin increases the performance of mitogen-activated protein kinase (MAPK), kinase (MEK) 3/6-p38 signaling pathways, and MAPK downstream from the AMPK cascade, which will in turn increase cellular glucose consumption [30, 31]. Curcumin stimulates adenosine monophosphate-activated protein kinase, which suppresses gluconeogenesis through preventing phosphoenolpyruvate carboxykinase and glucose-6 phosphatase [32, 33].

Probably, the anti-inflammatory property of curcumin is also effective in regulating glucose and lipid metabolism. As pro-inflammatory cytokines is higher in patients with PCOS [34]. TNF-α can stimulate serine phosphorylation of the insulin receptor, which leads to insulin resistance [35]. Curcumin significantly reduces TNF-α and interleukin-6 levels [36, 37]. Therefore, it can reduce insulin sensitivity and insulin resistance caused by obesity [38, 39]. Curcumin can upregulate the gene expression of peroxisome proliferator-activated receptor-gamma coactivator 1 alpha (PGC-1α), which rises the activity of glutathione peroxidase, reducing the oxidative stress expression [26].

Also, it seems that curcumin affects glucose metabolism by causing changes in enzyme activity associated with glucose; curcumin increases hepatic glucokinase activity while significantly decreasing glucose 6-phosphate and phosphoenolpyruvate carboxykinase activities. Curcumin stimulates the release of glucagon-like peptide-1 [40, 41]. The reason behind the differences between our study and other studies can be the differences in demography, age, genetics, anthropometrics, dosage, pharmacokinetic factors, follow-up duration, and sample size.

Following all principles of a clinical trial such as random allocation, hiding allocation, and blinding were among the strengths of this study. No loss of samples was another strength of this study. The small sample size was one of the limitations of this study and having a bigger sample size is recommended for further studies. Also, the study power was lower than 80% for detecting of significant difference between groups for testosterone level outcome.

## Conclusion

Curcumin decreased FBS levels and improved menstruation characteristics (amenorrhea, oligomenorrhea, and menstrual irregularities) in women with PCOS but did not affect other metabolic, hormonal, and hirsutism indices. More studies using a larger sample size are required for a definitive conclusion.

## Data Availability

The datasets generated and/or analysed during the current study are not publicly available due to limitations of ethical approval involving the patient data and anonymity but are available from the corresponding author on reasonable request.

## **Abbreviations**

FBI: Fasting Blood Insulin
FBS: Fasting Blood Sugar
LDL-C: Low Density Lipoprotein- cholesterol
HDL-C: High Density Lipoprotein- cholesterol
SHBG: Sex Hormone Binding Globulin
IRCT: Iranian Registry of Clinical Trials
PCOS: Polycystic ovary syndrome
DHEAS: dehydroepiandrosterone sulfate
CAH: Congenital Adrenal Hyperplasia
FFQ: Food Frequency Questionnaire
SD: Standard Deviation
MD: Mean Difference
95% CI: 95% Confidence Interval
AMPK: AMP-activated protein kinase
MAPK: Mitogen-Activated Protein Kinase
HOMA-IR: Homeostatic Model Assessment for Insulin Resistance.
